# Comparison of the Effect of Adding Different Levels of Zinc Chloride, Curcumin, Zinc Oxide Nanoparticles (Zano‐NPs), Curcumin Loaded on Zano‐NPs on Post‐Thawing Quality of Ram Semen

**DOI:** 10.1002/vms3.70091

**Published:** 2024-11-04

**Authors:** Fatemeh Omidi, Hadi Hajarian, Hamed Karamishabankareh, Leila Soltani, Mojtaba Dashtizad

**Affiliations:** ^1^ Department of Animal Science Faculty of Agricultural and Engineering Science Razi University Kermanshah Iran; ^2^ Department of Animal Science National Institute of Genetics and Biotechnology Tehran Iran

**Keywords:** cryopreservation, Curc‐co‐ZnO‐NPs, curcumin, ram, ZnO‐NPs

## Abstract

**Objective:**

This study looked at how different concentrations of curcumin (Curc), zinc chloride (ZnCl_2_), zinc oxide nanoparticles (ZnO‐NPs) and Curc loaded on ZnO‐NPs (Curc‐co‐ZnO‐NPs) in cryopreservation dilution affected the quality of ram sperm after thawing.

**Methods:**

ZnO‐NPs were synthesised using *Berberis vulgaris* leaf aqueous extract. Then, Curc was loaded on the ZnO‐NPs that had been synthesised. We used analytical methods to look at the composition, morphology and size of green synthesised ZnO‐NPs and Curc‐co‐ZnO‐NPs, including UV‐Vis, zeta potential, EDX, DLS, FE‐SEM and FT‐IR. Using a Tris‐base extender containing various concentrations of Curc, ZnCl_2_, ZnO‐NPs and Curc‐co‐ZnO‐NPs (0, 1, 10 and 100 µg/mL), semen samples from four rams were combined. Sperm motility, viability, DNA and plasma membrane integrity, total abnormalities and malondialdehyde (MDA) generation were all evaluated in treatment groups after thawing.

**Results:**

The results showed that adding 1 µg/mL of ZnO‐NPs and Curc‐co‐ZnO‐NPs significantly reduced the level of MDA and total abnormalities (*p *< 0.05). Additionally, following the freeze‐thawing procedure, the presence of 1 µg/mL of Curc‐co‐ZnO‐NPs in the diluent of ram sperm significantly increased the percentage of sperm viability and motility in comparison to the control and other treatment groups (*p* < 0.05). Furthermore, as compared to the control group and other treatments, treatments containing 1 µg/mL of Curc‐co‐ZnO‐NPs significantly improved membrane and DNA integrity (*p *< 0.05).

**Conclusions:**

It appears that following freeze‐thawing, the Curc‐co‐ZnO‐NPs (1 µg/mL) enhanced sperm parameters.

## Graphical abstract

1. In this study, ZnO‐NPs were synthesised using barberry leaves and identified with the aid of zeta potential, EDX, DLS, FE‐SEM and FT‐IR.

2. In the second phase of the study, nanoparticles were loaded with the aid of curcumin and identified by zeta potential, EDX, DLS, FE‐SEM and FT‐IR techniques.

3. In the next phase, different concentrations (ZnO‐NPs), Curc loaded on ZnO‐NPs (Curc‐co‐ZnO‐NPs), curcumin and zinc chloride were added to the diluted ram semen freezing extender.

4. After thawing, the concentration of 1 µg/mL of Curc‐co‐ZnO‐NPs had a better effect on maintaining sperm quality.

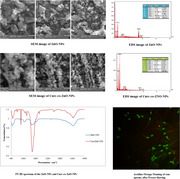

## 1 Introduction

Ram sperm is more vulnerable to cryopreservation than that of other mammalian species due to its polyunsaturated phospholipid membrane structure (Alcay et al. 2015). According to Amann and Pickett (1987), temperature fluctuations during freeze‐thawing procedures, the formation of ice crystals, changes in the plasma membrane and the production of reactive oxygen species (ROS) are among the other factors that can cause sperm damage during semen cryopreservation. These sperm damage factors may affect fertility and biological processes.

Antioxidants are added to the semen diluent to reduce the harmful effects of oxidative stress on the sperm (Aurich 2005). An imbalance between the generation and degradation of ROS can impair the functioning of sperm, leading to reduced membrane integrity, motility and fertility. Oxidative stress is considered one of the primary causes of sperm damage (Aitken et al. 2012; Soltani 2023). Antioxidants neutralise the intermediate chemicals produced during the energy generation process in the electron transport chain of cells. During the freeze‐thawing process, antioxidants help the spermatozoa resist the formation of ROS. Barzegar and Moosavi‐Movahedi (2011) have reported that curcumin (Curc) can help reduce the negative effects of oxidative stress on sperm. Curc has antioxidant properties and can scavenge free radicals, which makes it useful in increasing sperm motility in patients with asthenospermia (Zhou et al. 2020). Curc has also been found to enhance acrosome response and capacitation of sperm during both in vitro and in vivo fertilisation (Alizadeh et al. 2018). It has been shown to have positive effects on sperm cryopreservation in domestic animals such as cattle (Bucak et al. 2010), buffalo (Shahar et al. 2014) and boars (Chanapiwat and Kaeoket 2015).

Zinc (Zn) is a trace mineral that is vital to cellular functions. It acts as a co‐factor for DNA and RNA polymerases and more than 300 metallo‐enzymes that are involved in the metabolism of proteins, lipids, carbohydrates, DNA transcription and protein synthesis (Cummings and Kovacic 2009). Zinc is essential for the synthesis of testosterone and sperm development, which makes it critical for reproduction (Kvist, Björndahl, and Kjellberg 1987). It also helps maintain the membrane and DNA integrity of sperm (Prasad 2009). Adding zinc to a rooster semen extender can significantly reduce DNA damage and lipid peroxidation during sperm cryopreservation (Zhandi et al. 2020). In a 2012 study conducted by Kotdawala et al., it was demonstrated that zinc could positively affect maintaining and increasing molecular parameters. This includes DNA integrity, damage caused by freezing on mitochondrial function, fertilisation capacity and progressive mobility in frozen samples (Kotdawala et al. 2012).

Nanotechnology is a growing field of research in animal reproduction. Materials with a diameter of less than 100 nm, known as nanoparticles, have unique physical and chemical properties due to their small size. Ashtari et al. (2022) experimented to evaluate the protective effects of various zinc oxide nanoparticles (ZnO‐NPs) concentrations on sperm parameters after cryopreservation. Their findings suggest that 100 ppm of ZnO‐NPs can protect the pH, viability and motility of sperm after cryopreservation. Additionally, Khodaei‐Motlagh et al. (2022) reported that ZnO‐NPs had a positive impact on various sperm quality parameters, including motility, DNA and membrane integrity, viability and fertility rate, during cryopreservation of rooster semen.

In recent years, researchers have been working on improving the bioavailability and stability of Curc. They have made significant progress by using nanocarriers to overcome their limitations. Some studies have reported that Curc can be formulated into various nanoformulations, which can improve the drug's water solubility, bioavailability, stability, absorbability and overall therapeutic potential (Yallapu, Jaggi, and Chauhan 2013).

In comparison to organic drug carriers, inorganic nanomaterials such as graphene, carbon nanotubes, metallic nanoparticles, metal oxides and minerals offer several advantages. They are more functional and stable, with greater porosity and surface area. They excel at pharmaceutical loading, are more bioavailable, have fewer harmful side effects, can release medications in a controlled manner and are resistant to most organic solvents. As a result, they are preferred for drug delivery (Yavarpour‐Bali, Ghasemi‐Kasman, and Pirzadeh 2019; Kurien et al. 2007; Gomez et al. 2018; Jakubek et al. 2019; Salimi et al. 2024; Senapati et al. 2018; Naz et al. 2019).

The study explores the potential benefits of Curc loaded on ZnO‐NPs (Curc‐co‐ZnO‐NPs) during the process of freezing and thawing sperm. This study used barberry leaf extract to create ZnO‐NPs, which were then loaded with Curc. The properties of both the nanoparticles were then examined. The study assessed the advantages of different quantities of Curc, zinc chloride (ZnCl_2_), ZnO‐NPs and Curc‐co‐ZnO‐NPs on the post‐thawing quality of diluted ram semen. The aim was to determine the optimal concentration of different compounds that would improve the overall health of spermatozoa.

## 2 Materials and Methods

### 2.1 Chemicals

Sigma‐Aldrich Chemical Co., USA, provided the ingredients, including Curc, needed to prepare the extender.

### 2.2 Extract Preparation

Barberry plant leaves were collected from a garden of Kermanshah city. Leaves dried at a temperature of around 40°C, and a separate container was used to hold 20 g of dried *Berberis vulgaris* leaf, which was then ground into a powder using a mortar and combined with 100 mL of distilled water. Stir well and reheat in the microwave three times for 3 min at a power of 90 W. Following cooling, the filtrates were utilised as a reducing agent for the creation of nanoparticles after being filtered using Whatman filter paper No. 1, and to perform experiments, they were stored at 4°C (Behravan et al. 2019).

### 2.3 Synthesis of ZnO‐NPs

ZnO‐NPs were created using a green synthesis method involving *Berberis vulgaris* plant leaf extract. The process involved combining a 0.1 M aqueous zinc nitrate solution with 50 mL of distilled water and continuously stirring the mixture at room temperature. The resulting aqueous Zn(NO_3_)_2_ solution was then slowly added drop by drop to the 50 mL fruit extract at 90°C. The pH of the reaction was adjusted to 11 using 1N sodium hydroxide (NaOH). Shaking for 7 h. The resulting beige precipitate was separated by centrifugation, washed three times with distilled water, then one time with pure ethanol, and then dried in an oven at 70°C for 12 h. Subsequently, the beige powder was calcined for 2 h at 500°C in a muffle furnace (CWF, 1300, Carbolite, Derbyshire, UK). The result was a white powder that included ZnO‐NPs (Soltani, Samereh, and Mohammadi 2022).

### 2.4 Synthesis of Curc‐co‐ZnO‐NPs

This experiment involved mixing solutions of 1 mg/mL of Curc and 1 mg/mL of green synthesised ZnO‐NPs in methanol at equal proportions. The mixture was then centrifuged (7000 rpm for 20 min) to extract the Curc‐ZnO‐NPs after being agitated for 48 h at room temperature. A UV‐Vis spectrophotometer was used to analyse the supernatant collected from the centrifuge and determine the degree of conjugation effectiveness. The unconjugated drug's absorbance was found at 418 nm. By deducting the total amount of added drug from the amount of unconjugated drug in the supernatant, conjugation efficiency was determined. To find the drug's conjugation efficiency, the standard curve equation was employed.

$$\begin{array}{ccc} & & \mathrm{Conjugation}\;\mathrm{efficiency}=\left[\left(\mathrm{Total}\;\mathrm{Drug}-\mathrm{Unconjugated}\;\mathrm{Drug}\right)\right./\\ & & \;\left.\mathrm{Total}\;\mathrm{Drug}\right]\times 100\;\left(1\right) \end{array}$$

The drug content of an unknown sample was estimated using a straightforward, repeatable and dependable procedure that involved comparing the unknown to a set of standard samples with known concentrations. A calibration curve was created by plotting the drug's concentrations against absorbance (λmax 418 nm) to ascertain the quantity of Curc in the organic solution. The procedure uses methanol and the Beer‐Lambert law to determine the drug concentration, which ranges from 0.85 to 3.78 µg/mL. Calculations were made for a graph and line equations illustrating the linear reversion of the obtained concentration and absorbance data. Plotting of the Curc standard curve with a linear inversion was done in (Figure  6). In the standard graph, the associated coefficient value was close to 0.9882. It demonstrates that in concentrations between 0.85 and 3.78 µg/mL, the medication complies with the Beer‐Lambert rule (Shahabadi et al. 2021).

### 2.5 Characterisation of ZnO‐NPs and Curc‐co‐ZnO‐NPs

Zeta‐potential, Fourier transform infrared spectroscopy (FTIR), field emission scanning electron microscopy (FE‐SEM), x‐ray spectrometer (EDX), dynamic light scattering (DLS) and UV‐Vis spectroscopy were used to characterise ZnO‐NPs and Curc‐co‐ZnO‐NPs. ZnO‐NPs and Curc‐co‐ZnO‐NPs optical characteristics were investigated using a T80 spectrophotometer for UV‐Vis absorption and samples in quartz cuvettes. The produced ZnO‐NPs and Curc‐co‐ZnO‐NPs absorption spectra were observed at various wavelengths between 200 and 800 nm (Ankamwar et al. 2005). With the aid of Fourier transform infrared spectroscopy, the potential functional group of ZnO‐NPs and Curc‐co‐ZnO‐NPs was investigated (FTIR Bruker Alpha).

The material's morphology is determined using a high‐resolution scanning electron microscope (TESCAN MIRA3) fitted with an energy‐dispersive x‐ray spectrometer. Using a particle size analyser (Microtrac, Nanotrac wave II Q), the size of the produced NPs was determined using the dynamic light scattering technique using laser light. A zetasizer device was used to determine the sizes of ZnO‐NPs and Curc‐co‐ZnO‐NPs.

### 2.6 Evaluation of Cytotoxicity of Different Concentrations of ZnCl_2_, Curc, ZnO‐NPs and Curc‐co‐ZnO‐NPs

For the measurement of metabolic activity, the 3‐(4,5‐dimethylthiazol‐2‐yl)‐2,5‐diphenyltetrazolium bromide (MTT) test was used. 20 µL of MTT (5 mg/mL) and 200 µL of the sperm solution were mixed and then incubated for 2 h at 37°C with 5% CO_2_ (Mohammadi and Soltani 2021). Following this time, the formazan crystals generated by the viable spermatozoa reducing MTT were dissolved in 90 µL of dimethyl sulfoxide (DMSO), and the optical density was measured at 570 nm using a spectrophotometer.

A standard curve was constructed to calculate the number of viable cells. Aliquots of freeze‐killed and viable sperm were combined in ratios of 10:0, 8:2, 6:4, 4:6, 2:8 and 0:10 (*v*/*v*) to create the samples. Next, the MTT test was used to assess each sample's sperm viability. Then, MTT reduction rates were assessed both immediately and after a 2‐h incubation period at 37°C. A regression equation was used to represent the association between sperm viability and the MTT reduction rate (*Y* = 0.5666 ln(*x*) − 7.1794; *R* ^2^ = 0.98).

### 2.7 Animal and Semen Collection

Using an artificial vagina, four healthy adult Sanjabi rams (4 years old) provided semen samples twice a week for a total of twenty samples for the experiment. These samples were then pooled to prevent individual differences during the experiment and sent to the laboratory for initial assessments at 34°C. The samples selected for cryopreservation and the addition of compounds in this research met the following conditions: volume (0.75–2 mL), concentration (more than 3 × 10^9^ sperm per mL), motility (≈ 85%) and less than 10% abnormal sperm. The freezing extender used was Tris‐based and consisted of Tris 297.58 mM, citric acid 96.32 mM, fructose 82.66 mM and glycerol 6% (*v*/*v*) at a pH of 6.8 (Akhtarshenas et al. 2018). To dilute the semen until the concentration reached 200 million sperm/mL, 20% egg yolk was added to each semen pool in the Tris‐base extender. The samples were then chilled for 2 h to lower the temperature to 4°C and placed into 0.25 mL straws (IMV, L'Aigle, France). After chilling, the straws were held 4 cm above the liquid nitrogen for 10 min before being submerged in the liquid nitrogen for storage. For assessment, each straw was thawed individually for 30 sec at 37°C to evaluate the sperm (Akhtarshenas et al. 2018). The experimental treatments included thirteen groups: (1) 1 µg/mL of ZnCl_2_, (2) 10 µg/mL of ZnCl_2_, (3) 100 µg/mL of ZnCl_2_, (4) 1 µg/mL of Curc, (5) 10 µg/mL of Curc, (6) 100 µg/mL of Curc, (7) 1 µg/mL of ZnO‐NPs, (8) 10 µg/mL of ZnO‐NPs, (9) 100 µg/mL of ZnO‐NPs, (10) 1 µg/mL of Curc‐co‐ZnO‐NPs, (11) 10 µg/mL of Curc‐co‐ZnO‐NPs, (12) 100 µg/mL of Curc‐co‐ZnO‐NPs and (13) a group without antioxidant considering as the control group.

### 2.8 Evaluation of Sperm Motility

To evaluate total motility under a microscope, an aliquot of diluted cryopreserved spermatozoa treated with various antioxidants was placed onto a glass slide that had been warmed beforehand. 200 motile and immotile sperm were counted, and the proportion of the motile population was computed (Ghallab et al. 2017).

### 2.9 Sperm Viability

To evaluate the vitality of the sperm, we used the eosin‐nigrosin staining method (Evans and Maxwell 1987). A semen sample was spread onto slides after being diluted 1:2 with the staining solution (eosin‐Y 1.67 g, nigrosin 10 g, sodium citrate 2.9 g, dissolved in 100 mL distilled water). A phase‐contrast microscope (100×) was used to examine the smear after it had dried. Sperm cells (*n* = 200) were counted for both partially or partially stained (dead) and unstained (living) heads of sperm.

### 2.10 Functional Membrane Integrity

One method used to assess the sperm membrane's functional integrity was the hypo‐osmotic swelling test (HOST). A 100 mOsm hypo‐osmotic solution was combined with 30 µL of semen, and the mixture was incubated for 60 min at 37°C (Revell and Mrode 1994). Once the material was incubated, it was gently mixed. Smeared on a slide that had been preheated beforehand, a drop (15 µL) of the treated mixture was covered with a cover slip. 200 sperm were counted at 400× in at least five distinct microscopic fields. Next, it was noted what proportion of sperm cells had curved and enlarged tails (Buckett et al. 1997).

### 2.11 Sperm Morphology

The method of fixation liquid was used to determine the abnormal spermatozoon rate. The Hancock solution was used to fix the spermatozoon. Using phase‐contrast microscopy at 1000× magnification (Nikon Eclipse E600, Tokyo, Japan), at least 200 sperms per slide were analysed (Menon et al. 2011).

### 2.12 Sperm DNA Integrity

The integrity of the sperm DNA was evaluated using the Acridine Orange Staining technique. Each sample had 200 sperm counted using an epifluorescence microscope (480/550 nm). According to Mughal et al. (2013), spermatozoa with intact DNA (double‐stranded) glowed green, whereas those with damaged DNA (single‐stranded) shone red in fluorescence.

### 2.13 Measurement of Lipid Peroxidation (LPO) Levels

By employing the Malondialdehyde (MDA) Assay kit, the amount of LPO in spermatozoa was ascertained. Samples were mixed with reagents and cooked in a boiling water bath for an hour, resulting in the production of a pink colour. The following step involved pipetting the pink‐coloured supernatant into the microplate and measuring the absorbance at 532 nm to ascertain the MDA levels.

### 2.14 Statistical Analysis

Using SPSS version 16.0 software, we performed a one‐way analysis of variance and Duncan's multiple range tests on the frozen‐thawed semen characteristics data. Seven repetitions of the experiment were conducted, and the mean ± SD of the data was reported. A statistical significance level of *p* ˂ 0.05 was established.

## 3 Results

### 3.1 Field Emission Scanning Electron Microscopy

**FIGURE 1:**
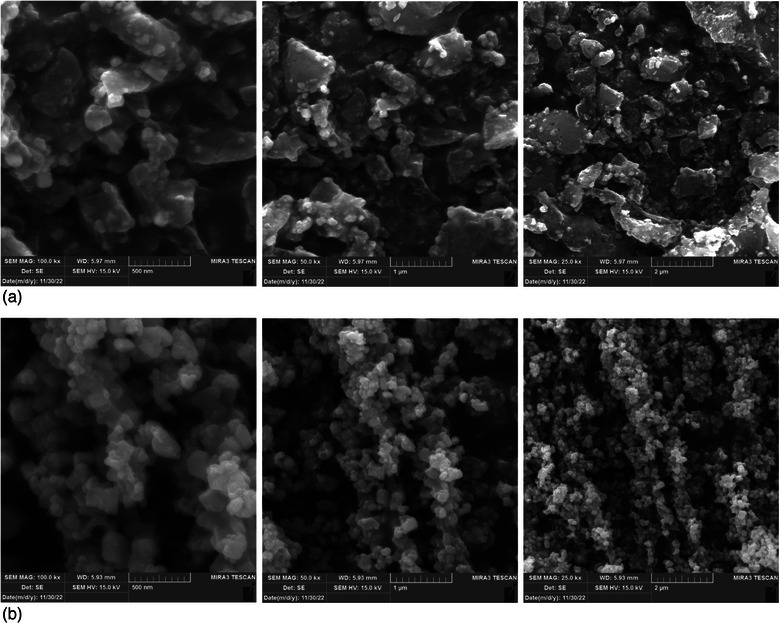
FE‐SEM images: (a) zinc oxide nanoparticles and (b) Curc‐co‐ZnO‐NPs.

The SEM images show that Curc‐co‐ZnO‐NPs form large clusters of particles. The successful loading of Curc onto ZnO‐NPs resulted in distinct nanostructures that drastically changed the shape of the ZnO‐NPs. The surface morphology of Curc‐co‐ZnO‐NPs and ZnO‐NPs was studied using a scanning electron microscope. Figure 1a, b illustrates the surface morphology of Curc‐co‐ZnO‐NPs and ZnO‐NPs at various magnifications. The SEM images clearly display the presence of large clusters of particles in the Curc‐co‐ZnO‐NPs.

### 3.2 Elemental Analysis (EDX)

**FIGURE 2:**
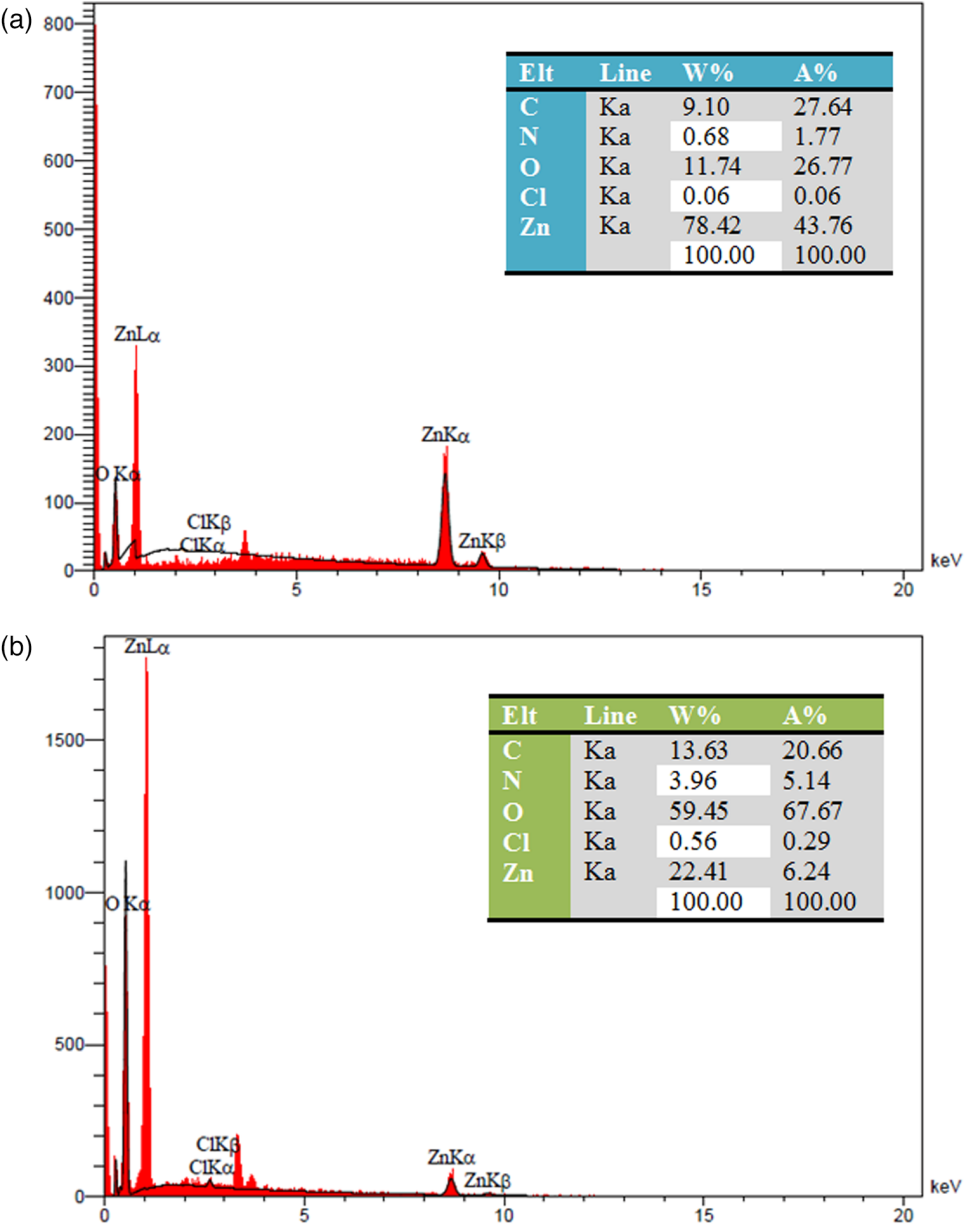
Elemental analysis of (a) ZnO‐NPs and (b) Curc loaded on zinc oxide nanoparticles. When referring to the electrons in a shell, the letters K, L and M indicate their value. K electrons are the closest to the nucleus and are *n* = 1 electrons. The size of the transition between shells is indicated by α and β. The transition from M to L or L to K is referred to as Lα or Kα, respectively, while the transition from M to K is called a Kβ transition. Additionally, the composition of nanoparticles can be described using parameters such as weight percentage (W%) and atomic percentage (A%).

The results of the elemental analysis of the synthesised ZnO‐NPs show the presence of zinc and oxygen signals, as validated in Figure 2a. The signal width of zinc atoms (78.42%), carbon atoms (9.10%), nitrogen atoms (0.68%), oxygen atoms (11.74%) and chlorine atoms (0.06%) can be observed in the elemental analysis of ZnO‐NPs produced by plant extract (green synthesis) (Figure 2a). These findings suggest that the synthesised nano‐powders have an exact elemental composition and high purity. The elemental analysis of Curc‐co‐ZnO‐NPs (Figure 2b) shows the signals for the elements zinc (22.41%), carbon (13.63%), nitrogen (3.96%), oxygen (59.45%) and chlorine (0.56%). No further peaks could be observed for other components. It is possible that the loading of Curc onto ZnO‐NPs caused a change in the quantity of signals.

### 3.3 Dynamic Light Scattering

**FIGURE 3:**
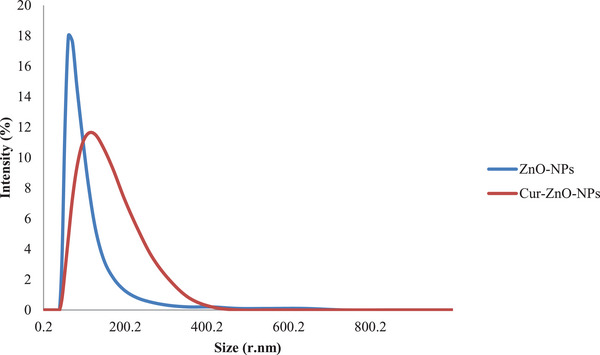
DLS spectrum of zinc oxide nanoparticles and Curc‐co‐ZnO‐NPs.

**FIGURE 4:**
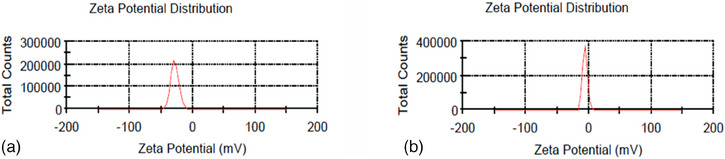
Zeta potential spectrum related to (a) zinc oxide nanoparticle and (b) Curc‐co‐ZnO‐NPs.

**FIGURE 5:**
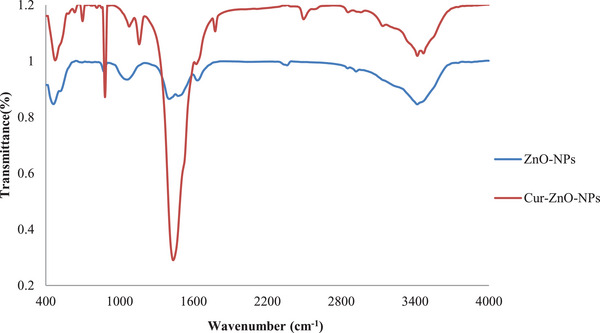
FT‐IR spectrum of the zinc oxide nanoparticle and Curc‐co‐ZnO‐NPs.

The DLS technique showed that the average hydrodynamic diameters of Curc‐co‐ZnO‐NPs and ZnO‐NPs were 135.7 nm and 88.68 nm, respectively (Figure 3). The dried powdered Curc‐co‐ZnO‐NPs can be stored for ≈ 1 month at room temperature without experiencing any deterioration or aggregation. This powder is both physically and chemically stable, easily dispersible in water and has a shelf life of one month (Pote et al. 2022). The decision to load Curc on ZnO‐NPs may be attributed to the size increase observed in the Curc‐co‐ZnO‐NPs compared to ZnO‐NPs (Figures 4 and 5).

### 3.4 Zeta Potential

Figure 4 shows the results of zeta potential when particles in a suspension have a significantly high negative or positive zeta potential value, they repel each other and nanoparticles do not come together. The magnitude of the zeta potential measures the potential stability of the colloid. If the particles have a low zeta potential, they will settle and aggregate. The zeta potential, or ZP, is determined by observing the speed of charged particles moving in the sample solution when an external electric field is present (Sapsford et al. 2011). Zeta potential readings typically range from +100 to −100 mV, and the magnitude serves as an indicator of colloidal stability. Zeta potential values greater than +30 mV or less than −30 mV indicate that nanoparticles are often quite stable. Conversely, smaller dispersions of zeta potential values may cause aggregation, coagulation or flocculation due to van der Waals interparticle interaction (Sapsford et al. 2011).

### 3.5 Fourier Transform Infrared Spectroscopy

The peaks at 698.42/cm and 880.91/cm are caused by the bending vibrations of the flavonoid and phenolic O─H groups, respectively. The Zn─O stretching vibrations can be observed in the peak at 476.04/cm. The peak at 2494.63 cm is small due to the connection with the C─H alkane. The minor absorption peak at 3418.45 cm indicates the O─H stretching vibrations, whereas the little peak at 1775.182 cm indicates the presence of primary amines. The sharp peak at 1434.61 cm signifies the C═O stretching vibrations in polyphenols, while the peak at 1158.86 cm represents the C═C stretching vibrations in the aromatic ring. The little peak at 1077.20 cm shows the stretching vibrations of the C─O molecule (Figure 5). These findings are consistent with other research studies (Mousa and Khairy 2020).

The peak at 1402.65 cm in the Curc‐co‐ZnO‐NPs sample indicates the stretching vibrations of the C═O ketone group. Similarly, the large peak at 1063.6 cm represents the stretching vibrations of the C─O group. The spectrum at 2852 cm is due to the O─H vibrations of the phenolic group. Upon loading Curc on ZnO‐NPs, the C═O stretching vibrations have decreased from 1434.61 cm to 1402.4 cm. The tiny yet wide absorption peak at 3417.93 cm indicates the O─H stretching vibrations.

### 3.6 UV‐Vis Spectrum

**FIGURE 6:**
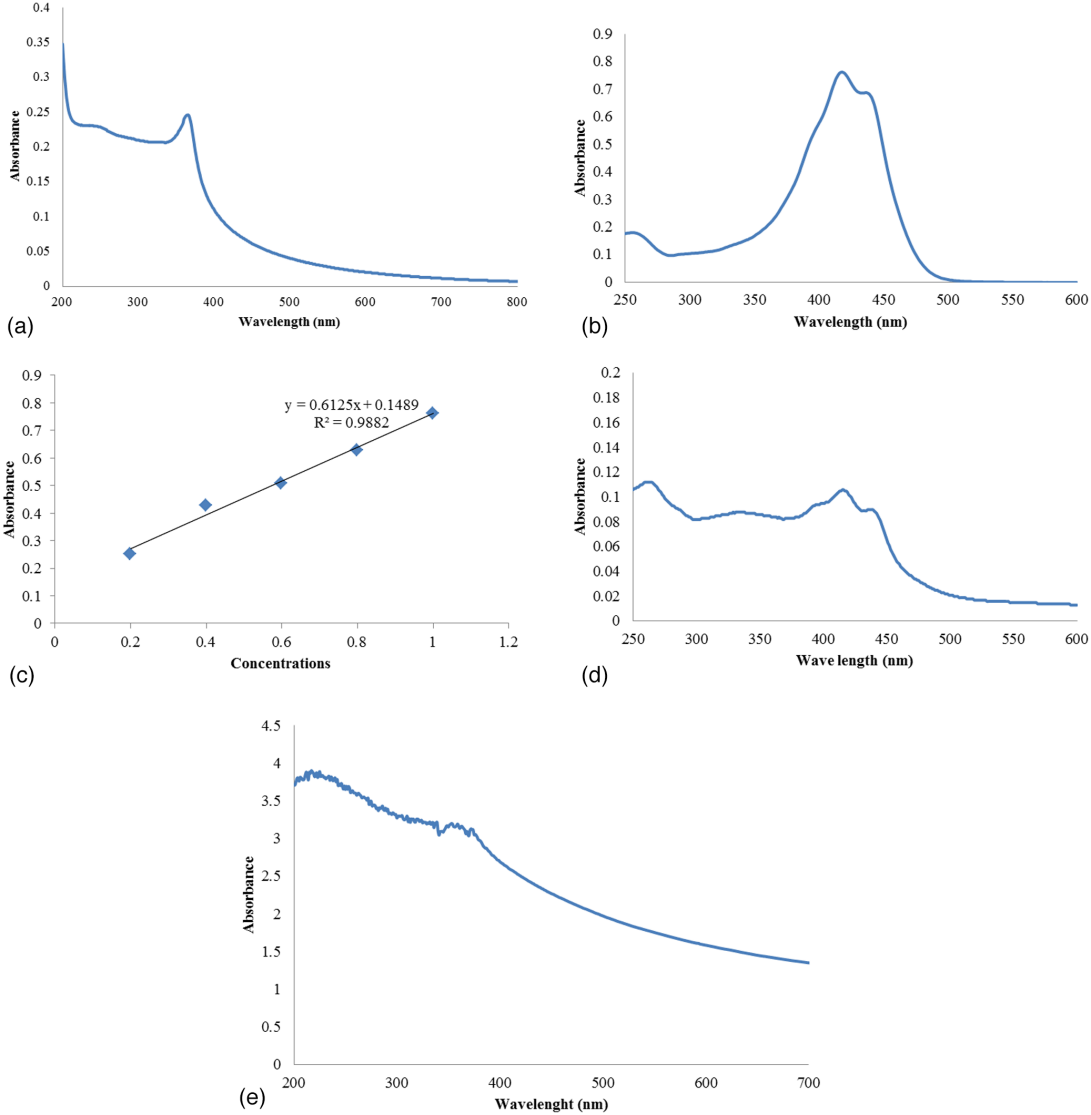
UV‐Vis spectrum of (a) ZnO‐NPs, (b) Curc, (c) Curc standard graph, (d) supernatant after Curc‐co‐ZnO‐NPs and (e) Curc‐co‐ZnO‐NPs.

The research focused on analysing ZnO‐NPs produced from plant extracts using UV‐Vis spectroscopy. The results indicated that the highest absorption of these nanoparticles occurred at 365 nm, as shown in Figure 6. To determine the molar absorption coefficient of the Curc, a standard graph was created, and various dilutions of the Curc were prepared to assess their absorption. Using a formula, the amount of Curc‐co‐ZnO‐NPs was then calculated. The absorption spectra of Curc (Figure 6) and ZnO‐NPs (Figure 6) at 329 nm were compared, and it was found that Curc was indeed loaded onto the ZnO‐NPs. The similarity between the absorption spectra of these two compounds was used to make this determination. Additionally, the standard graph was used to evaluate the amount of medicine loaded, which was determined to be 41.4%.

### 3.7 Analysing the Effects of Various Concentrations of ZnCl_2_, Curc, ZnO‐NPs and Curc‐co‐ZnO‐NPs on the Viability of Freshly Diluted Sperm Using the MTT Assay

Table 1 shows the results of a study on the viability of fresh sperm in ram semen diluent. The study examined how different concentrations of ZnCl_2_, Curc, ZnO‐NPs and Curc‐co‐ZnO‐NPs affect sperm viability when added to the diluent. According to the findings, the number of viable sperm was considerably greater in the ZnO‐NPs and Curc‐co‐ZnO‐NPs group when added to the ram semen extender at a concentration of 1 µg/mL than in the control and other treatment groups (*p* < 0.05). On the other hand, when 100 µg/mL of ZnCl_2_ and Curc were added, there were significantly fewer viable sperm in the ram semen diluent compared to all other treatment groups (*p* < 0.05). Overall, the study suggests that adding ZnO‐NPs and Curc‐co‐ZnO‐NPs at a concentration of 1 µg/mL to the ram semen extender may enhance sperm viability.

**TABLE 1:** The vitality of fresh ram sperm with the addition of ZnCl_2_, Curc, ZnO‐NPs and Curc‐co‐ZnO‐NPs at varying concentrations using the MTT assay.

Treatments	Number of viable cells
Control	548810.6 ± 3390.09^cd^
1 µg/mL of ZnCl_2_	541587.6 ± 955.85^ef^
10 µg/mL of ZnCl_2_	548324.7 ± 2903.22^cd^
100 µg/mL of ZnCl_2_	529337.1 ± 7440.26^e^
1 µg/mL of Curc	546718.3 ± 3896.26^de^
10 µg/mL of Curc	554649.6 ± 2447.26^b^
100 µg/mL of Curc	526507.8 ± 929.24^e^
1 µg/mL of ZnO‐NPs	565526.4 ± 3493.35^a^
10 µg/mL of ZnO‐NPs	553668.3 ± 1465.76^bc^
100 µg/mL of ZnO‐NPs	537779.3 ±1898.26^f^
1 µg/mL of Curc‐co‐ZnO‐NPs	565519.3 ± 499.04^a^
10 µg/mL of Curc‐co‐ZnO‐NPs	555632.9 ± 3432.23^b^
100 µg/mL of Curc‐co‐ZnO‐NPs	535887.2 ± 2837.37^f^

*Note*: There is no statistically significant difference (*p *> 0.05) between the means of the rows that share at least one letter.

### 3.8 The Impact on the Percentage of Total Sperm Motility and Viability by Adding Varying Doses of ZnCl_2_, Curc, ZnO‐NPs and Curc‐co‐ZnO‐NPs to Ram Semen Diluent After Freeze‐Thawing

Table 2 presents the impact on total sperm motility when different amounts of ZnCl_2_, Curc, ZnO‐NPs and Curc‐co‐ZnO‐NPs are added to ram sperm diluent during the freeze‐thaw process. After thawing, the group treated with Curc‐co‐ZnO‐NPs at a concentration of 1 µg/mL showed a significantly higher overall motility percentage compared to the control and other treatment groups (*p *< 0.05). While the overall motility quantity increased numerically (*p* > 0.05), there was no noticeable difference between the groups treated with 1 µg/mL of ZnO‐NPs and 1 µg/mL of Curc. Following the freeze‐thaw process, the diluent containing 100 µg/mL of ZnCl_2_ exhibited a significantly lower total motility percentage in ram semen compared to the other treatment groups (*p *< 0.05).

**TABLE 2:** Total motility and viability % of thawed‐frozen ram sperm following the addition of ZnCl_2_, Curc, ZnO‐NPs and Curc‐co‐ZnO‐NPs at varying doses.

Treatments	Total motility %	Viability %
Control	59.53 ± 0.57^f^	62.69 ± 0.38^f^
1 µg/mL of ZnCl_2_	60.91 ± 1.12^f^	63.87 ± 0.57^f^
10 µg/mL of ZnCl_2_	63.44 ± 1.73^de^	66.94 ± 1.47^de^
100 µg/mL of ZnCl_2_	44.90 ± 1.11^h^	46.89 ± 0.80^h^
1 µg/mL of Curc	62.46 ± 1.64^e^	65.99 ± 1.16^e^
10 µg/mL of Curc	65.18 ± 2.27^c^	69.38 ± 1.39^b^
100 µg/mL of Curc	49.21 ± 0.32^g^	51.87 ± 1.00^g^
1 µg/mL of ZnO‐NPs	67.55 ± 1.56^ab^	68.60 ± 1.40^bc^
10 µg/mL of ZnO‐NPs	64.78 ± 0.80^cd^	67.54 ± 1.43^cd^
100 µg/mL of ZnO‐NPs	49.97 ± 1.22^g^	51.84 ±1.06^g^
1 µg/mL of Curc‐co‐ZnO‐NPs	68.82 ± 1.12^a^	71.45 ± 1.62^a^
10 µg/mL of Curc‐co‐ZnO‐NPs	66.22 ± 1.43^bc^	67.31 ± 1.53^cde^
100 µg/mL of Curc‐co‐ZnO‐NPs	49.26 ± 1.07^g^	53.16 ± 1.24^g^

*Note*: There is no statistically significant difference (*p *> 0.05) between the means of the rows that share at least one letter.

In Table 2, the viability percentage is determined by the addition of different amounts of ZnCl_2_, Curc, ZnO‐NPs and Curc‐co‐ZnO‐NPs to ram semen diluent after freeze‐thawing. Adding 1 µg/mL of Curc‐co‐ZnO‐NPs to the ram semen extender resulted in a significantly higher (*p* < 0.05) percentage of viable sperm compared to the control and other treatment groups. On the other hand, ZnCl_2_ at a concentration of 100 µg/mL led to a significantly decreased percentage of viability (*p* < 0.05) in the ram semen diluent after the freeze‐thawing process, unlike the other treatment groups.

### 3.9 The Impact on the Percentage of Sperm Membrane and DNA Integrity by Adding Varying Doses of ZnCl_2_, Curc, ZnO‐NPs and Curc‐co‐ZnO‐NPs to Ram Semen Diluent After Freeze‐Thawing

Table 3 presents the findings regarding the impact on membrane integrity percentage of adding varying concentrations of ZnCl_2_, Curc, ZnO‐NPs and Curc‐co‐ZnO‐NPs to ram semen diluent following the freeze‐thawing process. When 1 µg/mL of Curc‐co‐ZnO‐NPs was added to the ram semen extender, the percentage of sperm membrane integrity was significantly higher (*p* < 0.05) compared to the control and other treatment groups. However, the addition of 100 µg/mL of ZnCl_2_ during the freeze‐thaw process resulted in a significantly lower percentage of membrane integrity compared to the control and other treatment groups (*p* < 0.05).

**TABLE 3:** The membrane and DNA integrity % of thawed‐frozen ram sperm following the addition of ZnCl_2_, Curc, ZnO‐NPs and Curc‐co‐ZnO‐NPs at varying doses.

Treatments	Membrane integrity percentage	DNA integrity percentage
Control	60.54 ± 2.55^f^	87.39 ± 0.87^e^
1 µg/mL of ZnCl_2_	63.24 ± 1.36^e^	87.77 ± 1.05^e^
10 µg/mL of ZnCl_2_	65.78 ± 2.18^bcd^	88.08 ± 1.69^de^
100 µg/mL of ZnCl_2_	45.25 ± 1.81^h^	81.60 ± 0.71^g^
1 µg/mL of Curc	63.95 ± 1.85^de^	87.60 ± 1.41^e^
10 µg/mL of Curc	64.94 ± 1.03^cde^	89.92 ± 1.26^cd^
100 µg/mL of Curc	48.44 ± 0.92^g^	83.30 ± 0.51^fg^
1 µg/mL of ZnO‐NPs	67.22 ± 1.29^b^	92.26 ± 1.44^b^
10 µg/mL of ZnO‐NPs	65.29 ± 2.18^bcde^	90.67 ± 0.24^bc^
100 µg/mL of ZnO‐NPs	49.78 ±1.41^g^	83.63 ±1.76^fg^
1 µg/mL of Curc‐co‐ZnO‐NPs	70.47 ± 2.53^a^	94.40 ± 0.40^a^
10 µg/mL of Curc‐co‐ZnO‐NPs	66.78 ± 2.17^bc^	91.59 ± 0.63^bc^
100 µg/mL of Curc‐co‐ZnO‐NPs	50.39 ± 1.91^g^	84.57 ± 1.78^f^

*Note*: There is no statistically significant difference (*p *> 0.05) between the means of the rows that share at least one letter.

After undergoing a freeze‐thaw process, Table 3 shows the percentage of intact DNA in ram sperm when different amounts of ZnCl_2_, Curc, ZnO‐NPs and Curc‐co‐ZnO‐NPs were used. The results indicated that DNA integrity significantly increased (*p* < 0.05) when 1 µg/mL of Curc‐co‐ZnO‐NPs was added, compared to the control and other treatment groups. Additionally, adding 100 µg/mL of ZnCl_2_ to the dilution after the freeze‐thaw process led to a significant increase in DNA integrity in ram semen. However, this effect was not observed in the treatment groups receiving Curc and ZnO‐NPs at a concentration of 100 µg/mL (*p* < 0.05).

### 3.10 The Impact on the Percentage of Total Sperm Abnormality and Production of MDA by Adding Varying Doses of ZnCl_2_, Curc, ZnO‐NPs and Curc‐co‐ZnO‐NPs to Ram Semen Diluent After Freeze‐Thawing

After subjecting ram sperm to the freeze‐thaw process and treating them with different amounts of ZnCl_2_, Curc, ZnO‐NPs and Curc‐co‐ZnO‐NPs, the results presented in Table 4 indicate the percentage of overall abnormalities. The findings reveal that adding 1 µg/mL of ZnO‐NPs and Curc‐co‐ZnO‐NPs significantly reduced the overall abnormality percentage (*p* < 0.05) compared to the other treatment groups. On the other hand, the ram semen diluent containing 100 µg/mL of ZnCl_2_ exhibited a significantly higher percentage of overall abnormalities (*p* < 0.05) compared to the other treatment groups.

**TABLE 4:** Total abnormality % and malondialdehyde production of thawed‐frozen ram sperm following the addition of ZnCl_2_, Curc, ZnO‐NPs and Curc‐co‐ZnO‐NPs at varying doses.

Treatments	Total abnormality percentage	Malondialdehyde production (nmol/mL)
Control	13.85 ± 1.36^b^	11.25 ± 0.01^e^
1 µg/mL of ZnCl_2_	12.93 ± 1.17^b^	9.63 ± 0.31^d^
10 µg/mL of ZnCl_2_	14.02 ± 0.71^b^	8.58 ± 0.03^b^
100 µg/mL of ZnCl_2_	23.02 ± 1.63^d^	13.06 ± 0.15^g^
1 µg/mL of Curc	13.68 ± 1.61^b^	9.24 ± 0.12^c^
10 µg/mL of Curc	12.79 ± 1.64^b^	8.59 ± 0.01^b^
100 µg/mL of Curc	17.44 ± 1.89^c^	13.34 ± 0.47^g^
1 µg/mL of ZnO‐NPs	10.55 ± 0.63^a^	7.77 ± 0.06^a^
10 µg/mL of ZnO‐NPs	13.21 ± 1.14^b^	9.11 ± 0.06^c^
100 µg/mL of ZnO‐NPs	18.42 ± 1.89^c^	12.05 ±0.25^f^
1 µg/mL of Curc‐co‐ZnO‐NPs	10.42 ± 1.23^a^	7.92 ± 0.04^a^
10 µg/mL of Curc‐co‐ZnO‐NPs	12.59 ± 1.47^b^	8.58 ± 0.01^b^
100 µg/mL of Curc‐co‐ZnO‐NPs	17.86 ± 1.72^c^	12.18 ± 0.25^f^

*Note*: There is no statistically significant difference (*p *> 0.05) between the means of the rows that share at least one letter.

In Table 4, the study presents the findings of MDA production during the freezing and thawing process of ram sperm. The study examined the effects of different concentrations of ZnCl_2_, Curc, ZnO‐NPs and Curc‐co‐ZnO‐NPs. It was found that the addition of ZnO‐NPs and Curc‐co‐ZnO‐NPs at 1 µg/mL substantially reduced the generation of MDA compared to the control and other treatment groups (*p *< 0.05). Conversely, the addition of 100 µg/mL of ZnCl_2_ and Curc to ram semen diluent during the freeze‐thawing process significantly increased the formation of MDA compared to the other treatment groups (*p* < 0.05).

## 4 Discussion

Arumugam, Subramaniam, and Krishnan (2021) synthesised ZnO‐NPs using *Berberis tinctoria* Lesch leaves and fruit extract. The nanoparticles were characterised using UV‐Vis and FT‐IR spectroscopy, which revealed distinct peaks at 274 nm, 467/cm and 456/cm. SEM analysis showed that the particles had a hexagonal shape, and EDX confirmed strong signals of Zn and oxygen. DLS results indicated that the NPs had sizes of 244 and 256 nm, with surface zeta potentials of −15.0 and −18.9 mV, respectively. Additionally, spherical ZnO‐NPs (20 nm) were produced using barberry extract, with −15.3 mV zeta potential and 28.8% antioxidant activity (Anzabi 2018).

Perera et al. (2020) conducted a study where they created ZnO‐NPs with different shapes and then studied the adsorption of Curc on the nanoparticles. Their research showed that Curc had a higher loading efficiency on ZnO‐NPs with long‐petal and javelin morphologies. However, they found no consistent pattern in the therapeutic performances of the nanocomposites, such as their antibacterial, anticancer and cytotoxic properties, based on the amount of Curc loaded. This suggests that factors other than the loading level, such as the shape and surface characteristics of nanoparticles, can impact therapeutic capabilities. The Curc was precipitated under ultrasonication to create a core‐shell Curc‐ZnO hybrid. The artificial core‐shell structure had a 45 nm ZnO core and a 12 nm Curc shell and demonstrated full water dispersibility. Compared to the commercial antibiotic amoxicillin, the core‐shell Curc‐ZnO nanocomposite demonstrated better antibacterial activity against *Staphylococcus pneumoniae* and *E. coli* (Varaprasad et al. 2019). A study showed that giving rabbits aflatoxin B1 (AFB1) along with Curc and ZnO‐NPs helped reduce the liver damage caused by the toxin. This was achieved by scavenging free radicals and activating antioxidant enzymes like glutathione peroxidase (GSH‐Px), catalase (CAT) and superoxide dismutase (SOD), demonstrating the hepatoprotective effects of ZnO and Curc (Atef et al. 2016).

It is suggested that optimising sperm preservation methods is a more practical alternative to standardised artificial insemination (AI) procedures in sheep species (Alvarez et al. 2019). One of the major challenges of cryopreservation is maintaining redox equilibrium, which can be disrupted by an increase in ROS and a reduction in antioxidant defence. Redox imbalance can negatively impact sperm quality, leading to decreased sperm motility, DNA structural damage and apoptosis (Alahmar 2019). To counteract the effects of oxidative stress during sperm freezing, antioxidant supplements are often used in various species (Dutta, Majzoub, and Agarwal 2019). Studies on stallions (Ghallab et al. 2017) and bulls (Eidan 2016) have shown that antioxidants can mitigate the harmful effects of oxidative stress caused by sperm cryopreservation. However, this is the first study to investigate the antioxidant effects of Curc‐co‐ZnO‐NPs during sperm freezing in ram.

The addition of 1 µg/mL of ZnO‐NPs and Curc‐co‐ZnO‐NPs to ram semen extender significantly enhanced the viability of fresh sperm compared to other treatment groups. This was demonstrated by the cytotoxicity test (MTT) used in the study. After 4 h of incubation, scientists found that supplementing the diluent with 0.6 µmol/mL Zn sulphate improved the motility of human sperm (Colagar, Marzony, and Chaichi 2009). Our study's findings are consistent with Barkhordari et al.’s research on the impact of ZnO‐NPs (10, 100, 500 and 1000 µg/L) on human sperm viability. The results indicate that the toxicity of ZnO‐NPs varies with concentration, with the maximum viability rate observed at concentrations of 10–500 µg/L and the highest cytotoxicity at 1000 µg/L of ZnO‐NP (Barkhordari et al. 2013). High Zn content in semen can negatively affect sperm quality metrics and limit oxygen absorption (Chohan, Griffin, and Carrell 2004).

The study found that frozen‐thawed sperms treated with a dilution containing 1 µg/mL of Curc‐co‐ZnO‐NPs had significantly higher percentages of viability and total motility compared to other groups (*p *< 0.05). The increase in sperm motility observed in this research can be attributed to the improvement in the activity of the sperm plasma membrane. It is evident that Curc, as shown in the study by Hamzavi et al. (2014), enhances the activity of mitochondria and ATP production. This, along with the improved action of the tail membrane, contributes to better sperm motility. After thawing, the percentage of viability and total motility in the treatments containing 100 µg/mL ZnCl_2_ decreased significantly compared to other treatment groups. A study also examined the use of ZnO‐NPs as an antioxidant in bull semen extender. The results indicated that samples with 1 µg/mL of ZnO‐NPs showed improvements in sperm motility, plasma membrane integrity and acrosome integrity after the freeze‐thaw process (Farhadi et al. 2022). Additionally, Ashtari et al.’s study found that ZnO‐NPs (40, 80 and 100 ppm) protected human sperm viability, motility and pH during the thawing process (Ashtari et al. 2022). In a research study, it was found that daily feeding of Curc to roosters resulted in improved integrity and function of the sperm plasma membrane. The researchers suggested that this improvement could be due to reduced oxidative stress and better sperm DNA integrity (Jalili et al. 2020).

In contrast to the current study, Fayez et al.’s (2023) research on supplementing diluted dog epididymal sperm with various concentrations of ZnO‐NPs showed that adding 100 µg/mL of ZnO‐NPs significantly increased sperm viability, motility, DNA, membrane and acrosome integrity and decreased MDA production compared to the control. Their findings contradicted the current study, which found that high concentrations of ZnO‐NPs were hazardous. The high concentrations reduced the viability, motility, DNA and membrane integrity and increased MDA of ram sperm after freeze‐thawing. It seems that different species of sperm react differently when nanoparticles are used as an antioxidant co‐factor. Also, the ability of spermatozoa to survive freezing and their sensitivity to cold temperatures can be attributed to the type of lipids present in the plasma membrane of the sperm. The ratio of omega‐3 to omega‐6 fatty acids, as well as the specific fatty acid profile, size and shape of spermatozoa, can differ between species, potentially impacting their response to cryopreservation (Hezavehei et al. 2018). 

Certain metallo‐enzymes, such as lactate dehydrogenase and sorbitol dehydrogenase, play a crucial role in increasing sperm motility. These enzymes may contain Zn in their structure (Dawei, Zhisheng, and Anguo 2010). Sperm motility is also influenced by protein kinase A (PKA) (Mizrahi and Breitbart, 2014). One study suggests that PKA activates Zn ions in sperm through the GPR39 receptor, which is linked to G protein (Michailov, Ickowicz, and Breitbart 2014).

The presence and activation of Zn^2+^ in the cell activates the adenylate cyclase enzyme. This enzyme converts ATP to cAMP (Michailov, Ickowicz, and Breitbart 2014). The increase in cAMP causes actin polymerisation (Etkovitz et al. 2007), which is necessary to control sperm motility (Itach et al. 2012). It is important to note that cAMP also leads to an increase in flagellar rate, resulting in hyperactive sperm motility (Shahar et al. 2014). Therefore, maintaining and enhancing sperm motility during the freeze‐thaw process is possible with an appropriate concentration of ZnO‐NPs.

Various studies have demonstrated the positive impact of Curc supplementation on sperm freezing diluent in different animal models, such as goats, bulls, mice and buffalos (Shah et al. 2016). Curc has also been found to improve the progressive motility and acrosome integrity of cryopreserved ram and bull sperm, particularly when exposed to oxidative stress (Omur and Çoyan 2016; Tvrdá et al. 2015). In human sperm cells, the addition of 20 µM of Curc as an antioxidant supplement to the freezing extender has been observed to increase motility and decrease the levels of ROS and DNA fragmentation after thawing (Santonastaso et al. 2021). Furthermore, diluents containing 2.5 mM Curc have been shown to improve sperm motility and DNA integrity, as well as preserve total antioxidant capacity (Soleimanzadeh and Saberivand 2013). In frozen bull sperm, it has been found to have positive effects on motility, morphology and antioxidant activity (Bucak et al. 2012).

Curc has high antioxidant properties due to the presence of two phenolic rings in the compound. It also contains other useful antioxidant groups, such as the β‐diketo group, carbon‐carbon double bonds and phenolic rings (Ak and Gülçin 2008). It's important to note that the core phenolic, β‐diketo group and methoxy functional groups of Curc work together to limit the oxidation of superoxide anion, which prevents the generation of hydroxyl radicals (Wright 2002). Additionally, Curc inhibits enzymes (such as lipoxygenase, cyclooxygenase and xanthine oxidase) that generate ROS and neutralise them. It also increases the activity of CAT, SOD and GSH‐Px enzymes in the cell. Curc is also effective in preventing heat shock and oxidative damage during freezing by stopping lipid peroxidation through scavenging free radicals (de Paula et al. 2006). According to Srinivas, Shalini, and Shylaja (1992), Curc has an eight‐fold greater ability than vitamin E to scavenge oxygen‐free radicals.

The potential benefits of Curc on sperm motility and viability are said to be due to its ability to eliminate free radicals, decrease oxidative stress and provide antioxidant qualities (Tvrdá et al. 2015). Additionally, Curc can inhibit human and mouse motility, capacity and performance by acidifying the intracellular pH of sperm and hyperpolarising the sperm cell membrane in a concentration‐dependent manner (Naz 2014). Our study showed that adding 1 µg/mL of ZnO‐NPs and Curc‐co‐ZnO‐NPs to the diluent significantly decreased the abnormality and the level of MDA, which is a byproduct of lipid peroxidation. This shows the beneficial effects of these compounds on the sperm membrane's lipid peroxidation process. Our findings are consistent with Farhadi et al.’s study, which revealed that the quantity of damaged DNA and MDA levels in sperm cells from Holstein bulls treated with 1 µg/mL of ZnO‐NPs significantly decreased to the control group (Farhadi et al. 2022). Enriching the diet of roosters with turmeric by‐products under normal conditions and heat stress led to an increase in sperm viability by reducing ROS production (Yan et al. 2017). However, a study on rabbits showed that adding turmeric to the diet did not improve the viability of fresh sperm (Ogbuewu et al. 2017). These differences could be due to factors such as the age of the animal, the species, the method of sperm processing (frozen or fresh) and the type of substance used (turmeric or Curc).

In a study that looked at how different amounts of Curc affect the quality of rooster sperm after the freezing process, researchers found that 30 µM of Curc demonstrated the highest level of membrane integrity and the lowest abnormality (Maroei et al., 2018). Similarly, in another study examining the impact of ZnO‐NPs on the human sperm cryopreservation extender, it was found that MDA and chromatin damage were significantly reduced (Isaac et al. 2017).

In a study, it was found that adding 1.0 mg/mL ZnO‐NPs to the citric acid‐fructose yolk extender enhanced the quality of Beetal Buck spermatozoa. This resulted in increased activities of endogenous antioxidant enzymes (SOD, CAT and peroxidases [POD]), improved structural and functional integrity and greater fertility (Khalique et al. 2023). Zn is known to enhance sperm's antioxidant capability, helping them combat oxidative stress caused by ROS (Afifi, Almaghrabi, and Kadasa 2015). It is important to note that Zn can compete with copper and iron for binding sites found in cell membranes. Substituting Zn for iron and copper may help avoid the production of very reactive radicals, as Zn is catalytically inert (Cruz, Oliveira, and do Nascimento Marreiro 2015; Oteiza 2012).

The research revealed that the group treated with 1 µg/mL of Curc‐co‐ZnO‐NPs exhibited significantly better membrane and DNA integrity. This was further supported by an experiment that investigated the effects of supplementing bull semen extender with ZnO‐NPs. The group given 1 µg/mL of ZnO‐NPs showed improved membrane and DNA integrity (Farhadi et al. 2022). Another study by Omur et al. also showed that adding 1 and 2 mM Curc to the diluent of ram sperm freezing extender resulted in the highest percentage of sperm with functional membrane integrity (Omur et al. 2014). These findings indicate that Zn has been shown to have a protective impact on membrane integrity in several studies (Afifi, Almaghrabi, and Kadasa 2015; Isaac et al. 2017). Zn has been demonstrated to have a protective effect on membrane integrity in several studies, while Curc may not phosphorylate a small proportion of calcium channels and sperm surface proteins. Tyrosine phosphorylation is important for sperm membrane functioning, capacitation or acrosome response and motility (Naz and Rajesh 2004).

Considering that the cytoplasmic content in sperm is low and is removed during the final stages of spermatogenesis, seminal plasma is a great defence against ROS by antioxidant enzymes such as SOD and CAT. The study by Amidi et al. (2016) demonstrated that supplementing the cryopreservation extender with CAT and SOD enhances in vitro sperm fertility (Amidi et al. 2016). On the other hand, during the studies, it was determined that the Zn element as a co‐factor for antioxidant enzymes in the extracellular environment can have a protective effect against oxidative stress by maintaining the activity level of CAT and SOD. On the other hand, Curc can increase the levels of intracellular glutathione, which is a phenolic compound and plays a role in many physiological actions of the cells, including cell protection from oxidative stress and protein and DNA production (Bucak et al. 2012). Research has shown that ROS can hinder the activity of important enzymes involved in ATP production and decrease the phosphorylation of sperm axon proteins (Saeed et al. 2016). As a result of this experiment, the researchers concluded that the observed enhancement in sperm motility in groups treated with Curc might be attributed to the reduction of ROS, increase in ATP production and improvement in axon protein phosphorylation. Finally, based on what was stated, despite the lack of investigation and evaluation of antioxidant enzymes such as CAT and SOD, according to the apoptosis assay and the evaluation of MDA production, which is known as an index of lipid peroxidation, it is believed that ZnO‐NPs coated with Curc can reduce the intensity of oxidative stress during the freeze‐thaw process and improve qualitative parameters by increasing the antioxidant capacity of semen and scavenging free radicals in sperm.

## 5 Conclusion

In this study, we synthesised Zn nanoparticles and coated them with Curc. We then investigated their different concentrations in the process of ram semen cryopreservation. We observed a positive effect of 1 µg/mL of Curc‐co‐ZnO‐NPs in reducing the amount of MDA and increasing the DNA integrity, which can improve the fertility rate of sperm after cryopreservation. We recommend adding ZnO‐NPs and Curc‐co‐ZnO‐NPs to ram sperm freezing diluent. Additionally, further studies on the molecular mechanisms of ZnO‐NPs, and ZnO‐NPs coated with specific antioxidants, especially Curc‐co‐ZnO‐NPs, are needed during semen cryopreservation. Furthermore, future studies are also necessary to explore the addition of these compounds in the process of in vitro fertilisation.

## Author Contributions

Hereby, we declare that all individuals contributed equally to this research in study design, data analysis and paper drafting.

## Ethics Statement

Animal husbandry and handling were conducted in accordance with the guidelines of Animal Ethics Committee (Permission number: IR.RAZI.REC.1402.050) of Razi University, Kermanshah, Iran.

## Conflicts of Interest

The authors declare no conflicts of interest.

### Peer Review

The peer review history for this article is available at https://publons.com/publon/10.1002/vms3.70091.

## Data Availability

The data that support the findings of this study are available from the corresponding upon reasonable request.
